# The Drinkers Degree: Risk Taking Behaviours amongst Undergraduate Student Drinkers

**DOI:** 10.1155/2015/965438

**Published:** 2015-12-02

**Authors:** Gillian O'Neill, Neil Martin, Jennifer Birch, Alison Oldam, Dorothy Newbury-Birch

**Affiliations:** ^1^Institute of Health and Society, Newcastle University, Newcastle upon Tyne NE2 4AX, UK; ^2^Durham County Council, Durham DH1 5UJ, UK; ^3^Balance, North East Alcohol Office, Durham DH1 1TW, UK; ^4^Student Wellbeing Service, Newcastle University, Newcastle upon Tyne NE1 7RU, UK; ^5^School of Health and Social Care, Teesside University, Middlesbrough TS1 3BA, UK

## Abstract

*Objective*. To examine risk taking behaviours associated with alcohol consumption amongst UK undergraduate students. Design and Methods. A cross-sectional web survey was used to assess attitudes and health behaviours. The survey included the Alcohol Use Disorders Identification Test (AUDIT). Students were also asked about why they drank alcohol; about their preferred alcoholic beverage; and if they had experienced any consequences associated with drinking alcohol as well as questions relating to sexual risk taking, drug use, and smoking.* Results*. 2779 (65% female; 84% White British) students completed some part of the survey. Of these, 98% (*n* = 2711) completed the AUDIT. Of the 92% that drank 66% (*n* = 1,643) were categorised as being AUDIT positive. 8% (*n* = 224) were categorised as probably alcohol dependent. Higher AUDIT scores were significantly associated with negative consequences such as unplanned sexual activity, physical injuries, and arguments. Other risk taking behaviours such as drug use and smoking were also found to be positively correlated with higher AUDIT scores; drug use; and smoking.* Conclusions*. The results from this study provide insight into students' alcohol consumption and associated risk taking. University policies need to protect students' overall health and wellbeing to ensure academic potential is maximised.

## 1. Introduction

University life brings with it the introduction to various social activities and new socialisation groups; this exposes students to various situations in which risk taking behaviour is deemed socially acceptable [1]. Student risky drinking and emotional distress have been hitting the media headlines [2] and research is identifying that students are drinking regularly above government recommended levels [3–5]. There is therefore a need to understand what universities can do to support students' wellbeing in this area [3, 4, 6]. During this transitional period in their lives, students become increasingly independent of their family and thus gain both responsibility and the potential to enable healthy personal and social development [7].

Risky drinking is a major public health problem and often equates to risk taking behaviour [8]. The impact of alcohol on the behaviour of young people has been well researched [9]. Adverse effects often arise from risky drinking which leads to intoxication and risk taking behaviour [10, 11]. Immediate problems result from accidents and trauma, physical and sexual assault including rape in young people, criminal behaviour including driving whilst intoxicated, and sexual risk taking [11–13].

Forty-five percent of men and 46% of women aged 16–24 in the general population report binge drinking (drinking more than twice the recommended daily amounts of 3-4 units per day for men or 2-3 units for women) [14]. Risky drinking definitions in the literature vary but for the purposes of this current research “binge drinking or risky drinking” was categorised as drinking more than 6 units of alcohol (one unit = 10 g ethanol) at any one drinking session for a woman and 8 units for a man. Risky drinking amongst undergraduates has been well documented with national data providing evidence that undergraduate students generally drink more than their nonstudent peers [3, 4, 15, 16]. A recent survey of undergraduates in seven universities in England, Wales, and Northern Ireland showed that the majority of students (76% of males, 65% of females) reported risky drinking at least once within the last two weeks, and problem drinking was prevalent in 20% of females and 29% of males [17]. Furthermore, UK university students have been shown to drink more than their European counterparts [1, 18].

The objective of this study was to examine risk taking behaviours associated with alcohol consumption amongst UK undergraduate university students and to discuss the findings in relation to the WHO Healthy University Framework.

## 2. Methods

### 2.1. Design and Participants

A cross-sectional web survey, adapted from the School Health Education Unit's (SHEU) Health Related Behaviour Questionnaire, was emailed to all undergraduate students registered with a university email account in one university in Northern England to assess attitudes and health behaviours [19]. A prize draw to win an Apple iPad was provided as an incentive to complete the survey.

This present study relates to the questions in the survey regarding alcohol, mental health, sexual health, smoking, and drug misuse. Demographic data was also collected on age, gender, ethnicity, living arrangements, type of course studied, and year of study.

Research suggests standardised alcohol screening tools, such as the Alcohol Use Disorders Identification Test (AUDIT) [20], are a highly sensitive and specific means of identifying current hazardous use of alcohol in adult populations, including college students [21–23]. Amongst adult drinkers, the AUDIT detects approximately 92% of genuinely excessive drinkers (sensitivity) and excludes approximately 94% of false cases (specificity) [24, 25] where a cut-off score of 8 or more (out of a possible score of 40) is used to detect hazardous use of alcohol and alcohol-related problems. Broken down further, respondents can be categorised as “abstainers” (zero); “lower risk” drinkers (1–7); at “increasing risk” (8–15); at “higher risk” (16–19); or “probably dependent” (20 and above). Increasing risk drinking implies a pattern of alcohol consumption that increases someone's risk of harm [26]. Higher risk drinking is defined as a pattern of alcohol consumption that is causing mental or physical damage [26]. Finally “dependent drinking” is described as a cluster of behavioural, cognitive, and physiological factors that typically include a strong desire to drink alcohol and difficulties in controlling its use [26]. Furthermore, students were also asked about why they drank alcohol; about their preferred alcoholic beverage; and if they had experienced any negative consequences associated with drinking alcohol.

In relation to mental health, questions were asked about whether students had experienced emotional or psychological problems (e.g., depression, anxiety, worry, or stress) that they felt interfered with their life; responses were never; yes more than 12 months ago; and yes within the last 12 months [19]. Moreover they were asked to determine whether a list of possible worries had affected them in the last month. Worries listed included study; money; alcohol; and drug use amongst other factors [19].

Questions were asked about whether contraception had been used on the last occasion that students had had sexual intercourse; whether emergency contraception had been used in the last 12 months; and whether the student or their partner had had a sexually transmitted infection in the last 12 months. Students were asked if they had ever smoked or currently smoked [19].

Questions related to drug use were based on suggestions from the local drug alcohol team. Students were asked to indicate whether they had ever used drugs; used them once or twice; used them regularly in the past but not now; currently used them less than once a week; and used them weekly or more often. Less than once a week and using weekly or more often were classified as current use. Cannabis, speed/amphetamine, cocaine, crack, acid/LSD, magic mushrooms, ecstasy, aerosol/glue/solvents, poppers, heroin, body-building steroids; nonprescribed sedatives or tranquilisers, nonprescribed antidepressants, and ketamine were included on the list of drugs as well as space to add others.

### 2.2. Governance, Accountability, and Ethics

The protocol for the study was reviewed by the local Primary Care Trust (PCT) research and development department and granted approval as an evaluation; therefore ethics was not applicable whilst the lead author was working there as part of her Public Health Training. Governance mechanisms were established through approval from the PCTs senior management group and a multidisciplinary steering group provided an overview and scrutiny role as a substitute for an approval by an ethics committee.

The web survey was piloted with members of the steering group and 30 representatives from the student union and amended based on feedback received. The survey was changed to include the ability to skip questions that students did not want to answer. The results from the pilot were not included in the final study. The pilot also indicated that students thought an incentive for completing the survey would be useful and therefore a prize draw for an iPad was included in the study. The survey was reviewed by senior managers of the university prior to dissemination.

## 3. Process

The survey was emailed to all students registered on campus' email addresses. The survey was live for three weeks from the end of April to the beginning of May 2012, just before exams began. An introductory email from the University Academic Registrar highlighted the purpose of the survey and emphasised that all responses would remain anonymous and confidential and that a final report would be collated at a group level. Contact information was provided at the end of the survey for local support services (including university support services) in case any sensitive issues had been raised whilst completing the questionnaire. One email reminder was sent midway through the three-week time period.

The questionnaire data was analysed using SPSS v.18. All responses were weighted by age, gender, and ethnicity to make the findings representative of the university population at the time of the survey. Statistically significant results were identified by using the 1% level, instead of the conventional 5%, due to large numbers of tests being run on the data. As AUDIT scores in the sample were positively skewed, nonparametric tests were used to look for statistically significant differences, an approach used in similar studies [4]. Differences in AUDIT score were identified using the Mann-Whitney *U* test (*Z* values) in the case of two groups or the Kruskal-Wallis analysis of variance by ranks (*χ* ^2^ values) in the case of more than two groups.

## 4. Results

Out of possible 14,814 undergraduate students registered with the university in 2011/12, 2779 (19%) completed the survey. Sixty-five percent (*n* = 1806) of respondents were female and 84% (*n* = 2,337) White British. The age profile of the survey respondents consisted of 5% (*n* = 145) 18 year olds, 20% (*n* = 550) 19 year olds, 23% (*n* = 649) 20 year olds, 23% (*n* = 624) 21 year olds, 23% (*n* = 647) 22–24 year olds, and 6% (*n* = 164) 25+ year olds. Eleven percent (*n* = 277) of those that answered the question reported current smoking (154; 12% males; 123; 9% females).

### 4.1. Representativeness

At a confidence level of 99% with a response rate of 19.2% and a sample of 2,842 from 14,812 there is a margin of error of ±1.7%. Due to the survey respondents differing in profile to the denominator student population the survey results were weighted for age, gender, and ethnicity (Table 1).

### 4.2. AUDIT Scores

In total 2711 (98%) students completed the AUDIT. Eight percent (*n* = 221) stated they were abstinent (7% (*n* = 97) males, 9% (*n* = 124) females). Of those that drank (scored more than zero on the AUDIT) the mean AUDIT score was 10.70; SD 6.16; range 1–39 (males 11.56; SD 6.37; range 1–39: females 9.80; SD 5.79; range 1–33). Of those that said they drank 66% (*n* = 1,643) were categorised as being AUDIT positive (score of 8+) (71% (*n* = 902) males, 61% (*n* = 741) females). Of those that drank, 8% (*n* = 224) of the overall sample were categorised as probably alcohol dependent (11% (*n* = 148) males, 6% (*n* = 76) females) (Table 2).

There were significant differences in mean AUDIT scores relating to the reasons why students drink alcohol (Table 3) “to feel good” (“strongly agree/agree” 11.12 (SD 6.27); “strongly disagree/agree” 9.28 (SD = 4.91); *Z* = −4.70, *P* < 0.0005), “to feel confident” (“strongly agree/agree” 11.17 (SD 6.19); “strongly disagree/agree” 8.67 (SD 5.58); *Z* = −6.21, *P* < 0.0005); “to get drunk” (“strongly agree/agree” 11.10 (SD 6.08); “strongly disagree/agree” 6.48 (SD 4.58); *Z* = −9.34, *P* < 0.0005); “to look cool” (“strongly agree/agree” 9.72 (SD 6.29); “strongly disagree/agree” 11.68 (SD = 5.79); *Z* = −7.92, *P* < 0.0005), and “because friends do” (“strongly agree/agree” 10.45 (SD 6.23); “strongly disagree/agree” 11.72 (SD 5.97); *Z* = −3.99, *P* < 0.0005).

Of those who stated they were abstinent (*n* = 221) a range of overlapping reasons were stated for this. Forty-five percent (*n* = 98) said that they did not drink for religious beliefs (53% (*n* = 50) of males and 39% (*n* = 48) of females), 36% (*n* = 78) said that they did not drink for lifestyle reasons such as fitness (44% (*n* = 42) of males and 29% (*n* = 36) of females), 47% (*n* = 102) did not like the taste of alcohol (37% (*n* = 35) of males and 55% (*n* = 67) of females), 2% (*n* = 5) had previous problems with alcohol and were now abstinent (2% of females), and 8% (*n* = 18) have experienced family problems through drinking and have chosen to be abstinent as a result (8% (*n* = 8) of males and 8% (*n* = 10) of females).

### 4.3. AUDIT by Gender, Age, and Ethnicity

Males had statistically significant higher AUDIT scores than females (males 11.56 (SD 6.37); females 9.80 (SD 5.79); *Z* = −6.45, *P* < 0.0005).

When looking at gender by age group, males had statistically significant higher mean AUDIT scores in some ages: 18 year olds (male 13.84 (SD 6.88); female 11.06 (SD 5.84); *Z* = −3.59, *P* < 0.0005), 20 year olds (male 11.89 (SD 5.91); female 9.83 (SD 5.83); *Z* = −4.88, *P* < 0.0005), and 21 year olds (male 11.21 (SD 5.94); female 9.09 (SD 5.05); *Z* = −3.73, *P* < 0.0005). When looking at gender by ethnicity AUDIT scores were significantly different amongst those classified as “White” (male 12.12 (SD 6.14); female 10.18 (SD 5.75); *Z* = −7.73, *P* < 0.0005).

There were significant differences in mean AUDIT scores between students of different ages (18s mean 12.46 (SD 6.52); 19s mean 11.86 (SD 6.59); 20s mean 10.84 (SD 5.95); 21s mean 10.21 (SD 5.63); 22–24s mean 9.03 (SD 5.54); 25+ mean 6.93 (SD 4.55); *χ* ^2^ = 133.01, df = 5, and *P* < 0.0005). Significant differences in age started between 19 and 20 year olds (*Z* = −3.05, *P* = 0.002) and for all age groups thereafter. There were also significant differences in mean AUDIT scores by ethnicity (“White” 11.12 (SD 6.03); “Chinese” 4.55 (SD 4.63); “other” 8.19 (SD 6.17); *χ* ^2^ = 142.86, df = 2, and *P* < 0.0005).

There was a significant overall difference in mean AUDIT scores of year of study with scores reducing as students' progress through university (first year 11.58 (SD 6.54); second year 10.76 (SD 6.08); third year 10.07 (SD 5.64); fourth year (or over) 8.54 (SD 5.29); *χ* ^2^ = 53.33, df = 3, and *P* < 0.0005). However, there were no significant differences in mean AUDIT score between students in their first and second years of study (*Z* = −2.54, *P* = 0.011) whilst significant differences existed between second, third, and fourth year (or over) of study (*χ* ^2^ = 25.62, df = 2, and *P* < 0.0005).

There was a significant difference in mean AUDIT scores of where students were currently living (“parental/family home” 8.25 (SD 4.86); “halls” 12.00 (SD 6.57); “privately rented” 10.43 (SD 5.89); “own home” 7.53 (SD 5.73); “other” 5.17 (SD 4.97); *χ* ^2^ = 106.07, df = 4, and *P* < 0.0005). There were significant differences in mean AUDIT scores between all combinations of residence except for students living in “parental/family home” and “own home” (*Z* = −1.38, *P* = 0.167) and “own home” and “other” (*Z* = −2.13, *P* = 0.034).

### 4.4. Negative Consequences and Worries Associated with Drinking Alcohol

Results showed significant differences between the mean AUDIT scores of students who had and had not experienced various negative consequences as a result of drinking. In all but one of the consequences listed, the mean AUDIT score was significantly higher in those students who had experienced the specific consequence. The only consequence not significant was that of “police caution or arrest for assault/affray” (Table 4).

There were also several significant differences between the mean AUDIT scores of students who worried about certain life issues. In descending order, some of the strongest significance of worry, as determined by the *Z* score, were “your alcohol use” (“often/most days” 19.44 (SD 6.25); “never/rarely/sometimes” 9.93 (SD 5.52); *Z* = −18.09, *P* < 0.0005), “your smoking” (“often/most days” 15.97 (SD 7.01); “never/rarely/sometimes” 10.49 (SD 6.02); *Z* = −7.94, *P* < 0.0005), “sex” (“often/most days” 13.24 (SD 6.93); “never/rarely/sometimes” 10.41 (SD 6.00); *Z* = −6.52, *P* < 0.0005), and “sexually transmitted infections” (“often/most days” 15.49 (SD 7.27); “never/rarely/sometimes” 10.56 (SD 6.06); *Z* = −6.10, *P* < 0.0005) (Table 5).

There were significant differences in mean AUDIT scores of students who “strongly disagreed/disagreed” and “strongly agreed/agreed” that the amount they drank at present was harmful to their health (“strongly disagreed/disagreed” 8.03 (SD 4.64); “strongly agreed/agreed” 16.32 (SD 5.99); *Z* = −25.64, *P* < 0.0005). There were also significant differences in mean AUDIT scores of students who “strongly disagreed/disagreed” and “strongly agreed/agreed” that the amount they drank at present was harmful to their studies (“strongly disagreed/disagreed” 9.20 (SD 5.15); “strongly agreed/agreed” 18.14 (SD 6.21); *Z* = −20.52, *P* < 0.0005).

### 4.5. Drug Use

Nine percent of students (*n* = 219) currently use any of the drugs listed in the survey (male *n* = 141; 11%, female *n* = 78; 6%). Of those that drank (scored more than zero on the AUDIT) (*n* = 219) the mean AUDIT score was 15.91, SD = 6.24, range 2–32 (male mean 16.10, SD = 6.25, range 2–32; female mean 15.55, SD = 6.24, range 5–26). There were significant differences between the mean AUDIT scores of student drinkers who did and did not currently use drugs (“did not currently use drugs” mean 10.24, SD = 5.90, range 1–39; *Z* = −12.33, *P* < 0.0005).

Six percent of students (*n* = 163) currently use cannabis (male *n* = 106; 8%, female *n* = 57; 4%). Of those that drank (*n* = 163) the mean AUDIT score was 15.71, SD = 6.38, range 2–32 (male mean 15.73, SD = 6.46, range 2–32; female mean 15.67, SD = 6.29, range 6–26). There were significant differences between the mean AUDIT scores of student drinkers who did and did not currently use cannabis (“did not currently use cannabis” mean 10.38, SD = 5.96, range 1–39; *Z* = −10.04, *P* < 0.0005).

### 4.6. Smoking

Students who smoked had significantly higher mean AUDIT scores than nonsmokers (“smoker” 14.17 (SD 6.60); “nonsmoker” 10.28 (SD 5.93); *Z* = −9.61, *P* < 0.0005).

### 4.7. Sex

Students who did not use any sort of contraception (or used the morning after pill) had significantly higher AUDIT scores than those who used some form of contraception the last time they had sex (“did not use” 12.50 (SD 6.58); “did use” 10.50 (SD 6.08); *Z* = −4.67, *P* < 0.0005). There were also significant differences in mean AUDIT scores of students who specifically used emergency contraception and those who did not (“did use” 13.05 (SD 6.06); “did not use” 10.76 (SD 5.80); *Z* = −7.20, *P* < 0.0005) and significant differences also existed between mean AUDIT scores of students who had contracted a sexually transmitted infection in the last year (“had a sexually transmitted infection [STI]” 14.83 (SD 7.29); “not had an STI” 11.04 (SD 5.87); *Z* = −4.99, *P* < 0.0005).

## 5. Discussion

This cross-sectional survey of risk taking behaviours associated with alcohol consumption amongst undergraduate students found, as other studies have, that students most likely consume alcohol at increasing risk levels or above are male, 18–20 year olds of a White British ethnicity, and living in halls in their first year of study [4, 27]. This study provides additional information on why students choose to drink alcohol. The social importance placed on alcohol by students to relax and boost confidence is of significant interest to university institutions as part of their duty of care to students.

The insight gained into some of the consequences experienced by students when drinking alcohol is of importance as it can affect students' general health and wellbeing. The significantly increased chances of being arrested for antisocial behaviour, having arguments with friends/family, having a physical injury (either resulting in attending hospital or not), and having unplanned sex make the student population who drink at higher levels more vulnerable to negative risky experiences which could have impacts on their chosen career paths and longer term health and wellbeing. This finding has also been found amongst students in previous research [12, 16].

So, the question is how these results can be used by universities to work towards changing behaviours. With more than 2.3 million students in the UK and approximately 370,000 staff, the university setting is an ideal context to embed health and wellbeing programmes and policies [28]. The World Health Organization's (WHO) Healthy University Framework has been in existence since the 1990s [7]. A healthy university “*aspires to create a learning environment and organisational culture that enhances the health, wellbeing and sustainability of its community and enables people to achieve their full potential*” [29]. It provides a framework to map the current health and wellbeing activities being delivered within a university and provides the mechanism to develop an improvement plan [29]. The results of this present study can provide a rich resource for understanding the student demography [30]. Recommendations can support university policy change as well as environmental and cultural shifts according to outcomes. The outcomes can also inform commissioning intentions and health service provision within the wider context.

The results of this present study provide insight into student drinking and positively correlated wider risk taking behaviours, which can inform university policies to protect students' health and wellbeing. The study university has considered the research results and has started to approach alcohol in a holistic and proactive manner. A multidisciplinary alcohol working group has been established to update the university alcohol policy in relation to staff and students. Numerous operational procedures have also changed. Since 2013 student enrolment documents have included core messages about the negative impact of alcohol and evidence based top tips to reduce these negative consequences. Also since 2013, Freshers' week was organised differently to ensure all promotional T-shirts did not advertise alcohol and that local pubs and clubs did not promote alcohol offers on campus grounds. The university offered increased financial assistance to societies on the basis that they did not take up sponsorship from external companies (most frequently bars and clubs who often hand out free alcohol). Discussions are taking place to use the study's finding in relation to the demographic profile of students drinking at the highest levels to develop a social marketing campaign to raise awareness of the impact risky alcohol consumption can have.

As with any study there are limitations. The survey response of 19% was low compared to other published electronic surveys [31] and is thus prone to low response bias. However email surveys in the student population have been found to have a similar completion rate to standard postal surveys [31]. As electronic surveys are cost-effective and timely it was deemed the best medium to use with this population group. There was no system built in to assess how many emails actually reached the student population. For various reasons including email accounts being full, or not being checked on a regular basis, the true denominator of the population reached is unknown as we do not know how many students actually read their email. As students receive hundreds of emails on a weekly basis, sent from a variety of sources, it is likely that the survey email may have been lost in the plethora of other mail, thus reducing the response rate [31]. Moreover, the study was conducted in a single university and so the results may not be generalizable to other university settings. Finally, although the AUDIT detects approximately 92% of genuinely excessive drinkers (sensitivity) and excludes approximately 94% of false cases (specificity) [24, 25] this is a self-report measure and not a diagnostic measure.

Finally, universities do not sit in isolation but are an integral part of the city within which they are based. The Healthy University Framework provides an opportunity to develop a multidisciplinary task group to review how a university can approach student alcohol consumption. Partnership working with local authority alcohol action groups can have an impact on pubs and clubs targeting students and promoting binge drinking. The study published here provides a case study example of how to gain an understanding of students' attitudes and behaviours towards alcohol and how a university can reduce the wider negative impact caused by risky consumption.

## Figures and Tables

**Table 1:** Student demographics.

Age Years	Ethnicity	Survey responses			Weighted responses			University population		
		Male	Female	Total	Male	Female	Total	Male	Female	Total
18	White	44	77	121	151	164	315	805	875	1680
	Chinese	1	4	5	3	3	6	14	18	32
	Other/not known	10	9	19	19	16	35	100	87	187

19	White	163	317	480	274	286	560	1461	1522	2983
	Chinese	5	8	13	8	11	19	45	56	101
	Other/not known	26	31	57	30	25	55	162	134	296

20	White	167	411	578	301	318	619	1603	1695	3298
	Chinese	4	16	20	12	18	30	65	98	163
	Other/not known	23	28	51	35	33	68	189	177	366

21	White	187	347	534	223	201	424	1190	1071	2261
	Chinese	8	21	29	14	13	27	77	68	145
	Other/not known	19	42	61	31	28	59	167	149	316

22–24	White	174	316	490	159	150	309	849	797	1646
	Chinese	20	30	50	18	13	31	98	70	168
	Other/not known	50	57	107	40	28	68	214	151	365

25+	White	56	78	134	63	45	108	334	240	574
	Chinese	2	0	2	4	0	4	22	20	42
	Other/not known	14	14	28	23	13	36	123	68	191

	Total *n*	**973**	**1806**	**2779**	**1408**	**1365**	2773^*∗*^	**7518**	**7296**	**14,814**
	Total %	**35.0%**	**65.0%**		**50.8%**	**49.2%**		**50.7%**	**49.3%**	

^*∗*^This number does not include the number who did not state their ethnicity.

**Table 2:** Mean (SD) and range of AUDIT scores weighted by age, gender, and ethnicity.

Age	*N*		AUDIT		Low risk		Increasing risk		Higher risk		Probably dependent		AUDIT positive	
					(0–7)		(8–15)		(16–19)		(20+)		(8–40)	
		Mean	SD	Range	*N*	%	*N*	%	*N*	%	*N*	%	*N*	%
Male drinkers														
18	158	13.84	6.88	1–39	32	20.4	66	41.5	34	21.7	26	16.4	126	79.7
19	288	12.51	6.77	1–32	72	24.8	124	42.9	46	16.0	47	16.3	217	75.3
20	316	11.89	5.91	1–27	81	25.6	154	48.6	42	13.4	39	12.3	235	74.4
21	241	11.21	5.94	1–32	65	26.8	125	51.8	30	12.6	21	8.9	176	73.0
22–24	187	9.91	5.97	1–27	75	40.0	80	42.5	19	10.2	14	7.3	113	60.4
25+	82	7.3	4.72	1–22	46	56.6	30	36.5	4	5.5	1	1.4	35	42.7
Total	1272	11.56	6.37	1–39	370	29.1	577	45.4	177	13.9	148	11.6	902	70.9

Female drinkers														
18	158	11.06	5.84	1–33	46	29.1	78	49.5	25	16.0	9	5.4	112	70.9
19	294	11.21	6.36	1–30	94	32.0	125	42.7	40	13.7	34	11.6	199	67.7
20	333	9.83	5.83	1–33	128	38.5	158	47.4	26	7.8	21	6.3	205	61.6
21	215	9.09	5.05	1–26	88	41.0	99	46.2	19	9.0	8	3.8	126	58.6
22–24	167	8.06	4.84	1–31	85	50.8	71	42.2	8	4.5	4	2.5	83	49.7
25+	52	6.34	4.25	1–19	36	70.3	13	25.3	2	4.5	0	0	15	28.8
Total	1217	9.8	5.79	1–33	477	39.2	544	44.7	121	9.9	76	6.2	741	60.9

All drinkers														
18	316	12.46	6.52	1–39	78	24.7	143	45.5	60	18.9	34	75.0	237	75.0
19	582	11.86	6.59	1–32	166	28.4	249	42.8	86	14.8	81	71.5	416	71.5
20	648	10.84	5.95	1–33	209	32.2	311	48.0	68	10.5	60	67.7	439	67.7
21	456	10.21	5.63	1–32	153	33.5	224	49.2	50	10.9	30	66.7	304	66.7
22–24	355	9.03	5.54	1–31	160	45.1	150	42.3	27	7.5	18	54.9	195	54.9
25+	133	6.93	4.55	1–22	82	61.9	43	32.2	7	5.1	1	38.3	51	38.3
Total	2490	10.7	6.16	1–39	848	34.0	1121	45.0	297	11.9	224	65.9	1642	65.9

**Table 3:** Reasons for drinking alcohol.

Reasons for drinking alcohol	Strongly agree/agree			Strongly disagree/disagree			Mann-Whitney	
	*N*	Mean	SD	*N*	Mean	SD	*Z* score	*P* value
To feel good	1787	11.12	6.27	254	9.28	4.91	−4.7	<0.0005
To feel confident	1991	11.17	6.19	192	8.67	5.58	−6.21	<0.0005
To relieve stress	1877	10.81	6.15	207	11.11	6.38	−0.27	0.785
To feel relaxed	1944	10.73	6.11	177	10.75	5.88	−0.72	0.474
To look cool	990	9.72	6.29	1010	11.68	5.79	−7.92	<0.0005
To get drunk	2161	11.10	6.08	124	6.48	4.58	−9.34	<0.0005
Because friends do	1607	10.45	6.23	441	11.72	5.97	−3.99	<0.0005

**Table 4:** Differences in AUDIT score in relation to negative consequences of drinking.

Negative consequence as a result of alcohol consumption	Have experienced			Have not experienced			Mann-Whitney	
	*N*	Mean	SD	*N*	Mean	SD	*Z* score	*P* value
Hangover	1589	12.68	5.73	815	6.93	5.04	−24.59	<0.0005
Memory loss	1275	14.04	5.6	1129	6.98	4.29	−31.16	<0.0005
Physical injury, nonhospital attendance	508	16.48	5.73	1896	9.18	5.27	−23.82	<0.0005
Physical injury, hospital attendance	115	19.07	5.97	2289	10.31	5.84	−13.52	<0.0005
Alcohol poisoning, hospital attendance	16	17.00	6.53	2388	10.69	6.12	−3.95	<0.0005
Caution/arrest for ASB	29	21.83	7.46	2375	10.59	6.00	−7.25	<0.0005
Caution/arrest for assault	3	16.78	5.26	2400	10.72	6.14	−1.66	0.097
Victim of assault	57	18.53	6.74	2347	10.54	6.00	−8.17	<0.0005
Victim of sexual assault	15	15.93	7.09	2389	10.69	6.12	−3.16	0.002
Unplanned sex	327	15.76	5.97	2077	9.93	5.78	−16.07	<0.0005
Problems with partner/family/friends	137	17.36	7.21	2267	10.33	5.83	−11.3	<0.0005
None of the above	573	5.38	3.64	1831	12.4	5.79	−27.26	<0.0005

**Table 5:** Differences in AUDIT score in relation to worries.

How often have you worried about the things listed below in the last month?	Often/most days			Never/rarely/sometimes			Mann-Whitney	
	*N*	Mean	SD	*N*	Mean	SD	*Z* score	*P* value
Study, workload problems	1413	10.56	6.18	1073	10.90	6.12	−0.89	0.375
Money problems	763	11.50	6.63	1722	10.34	5.90	−4.1	<0.0005
Physical health	484	11.85	7.17	1996	10.43	5.84	−3.49	<0.0005
Emotional health	473	10.72	6.92	2014	10.70	5.97	−0.99	0.325
Problems with friends	253	11.19	6.92	2233	10.65	6.07	−1.08	0.281
Problems with lecturers and teachers	93	12.63	8.14	2392	10.63	6.06	−1.56	0.119
Boyfriend/girlfriend problems	385	12.07	6.73	2101	10.45	6.02	−4.62	<0.0005
Sex	260	13.24	6.93	2225	10.41	6.00	−6.52	<0.0005
Family problems	287	11.44	7.08	2200	10.60	6.02	−1.41	0.158
The way you look	777	11.56	6.50	1712	10.31	5.96	−4.07	<0.0005
The amount you are eating	844	11.68	6.69	1645	10.20	5.81	−4.92	<0.0005
What people think of you	750	11.68	6.66	1738	10.28	5.88	−4.29	<0.0005
Sexually transmitted infections	76	15.49	7.27	2410	10.56	6.06	−6.1	<0.0005
Your gambling, for example, lottery tickets	43	17.42	9.38	2442	10.59	6.02	−4.78	<0.0005
Your smoking	98	15.97	7.01	2389	10.49	6.02	−7.94	<0.0005
Your alcohol use	201	19.44	6.25	2284	9.93	5.52	−18.09	<0.0005
Your drug use	26	17.75	7.16	2459	10.62	6.10	−4.89	<0.0005
